# *Gardnerella* Exposures Alter Bladder Gene Expression and Augment Uropathogenic *Escherichia coli* Urinary Tract Infection in Mice

**DOI:** 10.3389/fcimb.2022.909799

**Published:** 2022-06-16

**Authors:** Nicole M. Gilbert, Valerie P. O’Brien, Chevaughn Waller, Ekatherina Batourina, Cathy Lee Mendelsohn, Amanda L. Lewis

**Affiliations:** ^1^ Department of Pediatrics, Division of Infectious Diseases, Washington University in St. Louis School of Medicine, St. Louis, MO, United States; ^2^ Human Biology Division, Fred Hutchinson Cancer Research Center, Seattle, WA, United States; ^3^ Department of Urology, Columbia University Irving Medical Center, New York, NY, United States; ^4^ Department of Obstetrics, Gynecology and Reproductive Sciences, University of California, San Diego, San Diego, CA, United States

**Keywords:** urobiome, bladder, dysbiosis, urothelium, bacterial vaginosis, urinary tract infection, RNA-seq

## Abstract

The anaerobic actinobacterium *Gardnerella* was first isolated from the bladder by suprapubic aspiration more than 50 years ago. Since then, *Gardnerella* has been increasingly recognized as a common and often abundant member of the female urinary microbiome (urobiome). Some studies even suggest that the presence of *Gardnerella* is associated with urological disorders in women. We recently reported that inoculation of *Gardnerella* into the bladders of mice results in urothelial exfoliation. Here, we performed whole bladder RNA-seq in our mouse model to identify additional host pathways involved in the response to *Gardnerella* bladder exposure. The transcriptional response to *Gardnerella* reflected the urothelial turnover that is a consequence of exfoliation while also illustrating the activation of pathways involved in inflammation and immunity. Additional timed exposure experiments in mice provided further evidence of a potentially clinically relevant consequence of bladder exposure to *Gardnerella*—increased susceptibility to subsequent UTI caused by uropathogenic *Escherichia coli*. Together, these data provide a broader picture of the bladder’s response to *Gardnerella* and lay the groundwork for future studies examining the impact of *Gardnerella* on bladder health.

## Introduction

*Gardnerella* comprises a genus of Gram-variable Actinobacteria that are frequently present in the microbiota of the female urogenital system. *Gardnerella vaginalis* has historically been regarded as a vaginal organism because it was first identified in vaginal fluid, where it was implicated as the causative agent in the prevalent condition clinically recognized as bacterial vaginosis (BV) (Leopold, 1953; Gardner and Dukes, 1954; Schwebke et al., 2014; Morrill et al., 2020). BV is a state of the vaginal microbiome that is composed of a polymicrobial mixture of anaerobic bacteria. Further studies over the past 50 years have revealed that *G. vaginalis* is frequently found among the vaginal microbiota outside the context of symptomatic BV (Krohn et al., 1989; Briselden and Hillier, 1990). However, even in asymptomatic women, *Gardnerella* is more frequently present and is often the predominant organism in the context of a vaginal “community state type” (CST) that is composed of a polymicrobial mixture of anaerobic bacteria (Ravel et al., 2011). *G. vaginalis* has a complicated taxonomic history, originally being named *Haemophilus vaginalis* and then *Corynebacterium vaginale* (Gardner and Dukes, 1955; Zinnemann and Turner, 1963). Most recently, a split of the *Gardnerella* genus into 13 distinct species has been proposed (Vaneechoutte et al., 2019). Some strains that have been characterized and previously referred to in the literature as *G. vaginalis* would fall into a different species with the newly proposed nomenclature, including the strain we used in this study. For ease of understanding, here, we primarily use only the genus name *Gardnerella.*

Urine is the second most common source of isolation of *Gardnerella*, after the vagina. *Gardnerella* is a rare cause of symptomatic urinary tract infection (UTI), and it is more often detected in urine samples collected in studies aimed at profiling the composition of the urinary microbiome, or “urobiome” (Kline and Lewis, 2016). In the first report of isolation from the bladder in 1968, *Gardnerella* was cultured from 159/1000 suprapubic aspirates from healthy pregnant women (Mcfadyen and Eykyn, 1968). Subsequent culture-based studies isolated *Gardnerella* in bladder aspirates from women with and without current or prior urinary tract diseases (Birch et al., 1981; McDowall et al., 1981; McDonald et al., 1982; Fairley and Birch, 1983; Gilbert et al., 1986). In studies using modern 16S sequencing and expanded quantitative culture methods, *Gardnerella* has emerged as one of the most frequently isolated members of the female urobiome and is the dominant organism in many women (Hilt et al., 2014; Pearce et al., 2014; Pearce et al. 2015; Gottschick et al., 2017; Jacobs et al., 2017; Price et al., 2020). Like *Gardnerella*, a majority of other urobiome bacteria are members of genera historically regarded as vaginal organisms, such as *Lactobacillus*. Recent studies have found substantial overlap between the urinary and vaginal microbiomes present concurrently in the same woman (Komesu et al., 2019; Brown et al., 2021; Hugenholtz et al., 2022). Although the presence of *Gardnerella* in urine specimens could reflect periurethral or vaginal colonization, the fact that many studies have cultured *Gardnerella* from urine collected directly from the bladder by suprapubic aspiration or catheterization strongly suggests that *Gardnerella* gains access to the bladder, at least transiently, in some women.

Whether, or how, urobiome members such as *Gardnerella* stably colonize the bladder remains to be determined. We also know very little regarding how the bladder responds to bacterial exposures outside of the context of symptomatic UTI. This is important because there has been growing interest in the concept of manipulating the urobiome as a therapeutic strategy for a wide range of lower urinary tract conditions (Jung and Brubaker, 2019; Cole et al., 2021; Jones et al., 2021; Garofalo et al., 2022). Mouse models have proven valuable in advancing our understanding of bladder responses to established uropathogens, but animal models examining common urobiome bacteria are limited. We previously developed a mouse model of *Gardnerella* bladder exposure (Gilbert et al., 1986; O'Brien et al., 2020). We refer to this model as an “exposure” rather than an infection because *Gardnerella* is cleared from the urinary tract within 12 h. Even such a transient presence of *Gardnerella* in the urinary tract was sufficient to trigger apoptosis and exfoliation of the superficial bladder epithelial (urothelial) cells (Gilbert et al., 2017). Exfoliation is an innate host response known to occur during symptomatic UTI that is presumably aimed at helping eliminate bacteria from the bladder *via* shedding of infected epithelial cells (Mysorekar and Hultgren, 2006; Lin et al., 2015). Here, we further probed the host response to *Gardnerella* in the urinary tract by examining the bladder transcriptome using RNA-seq. Two successive exposures to *Gardnerella* activated genes and pathways in the bladder that are related to DNA damage, programmed cell death, cell differentiation, and proliferation, which are consistent with the processes of urothelial exfoliation and renewal. Additionally, *Gardnerella* exposure influenced gene sets related to immune and inflammatory responses. Finally, we demonstrate that preexposure to *Gardnerella* resulted in heightened bacterial loads upon subsequent uropathogenic *Escherichia coli* (UPEC) experimental UTI, promoting persistent UPEC bacteriuria and increased bladder tissue titers. These findings provide proof of concept that even transient *Gardnerella* bladder exposures affect the bladder mucosa in ways that can alter the course of UPEC UTI.

## Results

### Effect of *Gardnerella* Exposures on the Bladder Transcriptome

**Figure 1:**
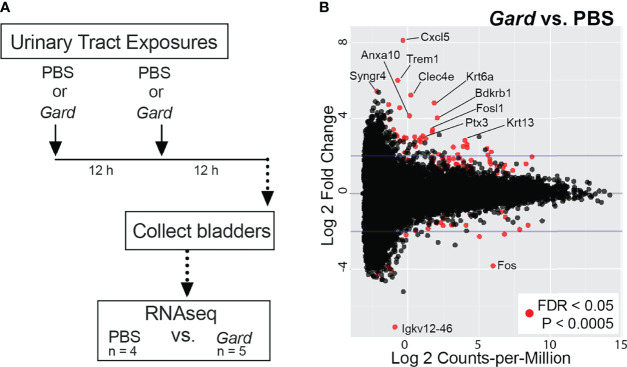
*Gardnerella* exposures alter the bladder transcriptome. **(A)** Schematic of the urinary tract exposure model used for RNA-seq. Female C57BL/6 mice were given intravesical “urinary tract exposures” of either PBS or *Gardnerella via* transurethral catheterization. Each mouse received two exposures that were administered 12 h apart. Bladders were collected 12 h after the second exposure and processed for RNA-seq. *Gardnerella*-exposed bladders were compared to PBS-exposed control bladders to identify differentially expressed genes and for gene set enrichment analyses. **(B)** MA-plot indicates genes that had altered expression in *Gardnerella* compared to PBS bladders. Red dots denote genes that were significantly differentially expressed after false discovery rate (FDR) correction for multiple comparisons. Genes discussed in the text are indicated by name.

We performed RNA-seq on whole bladders to identify host responses to *Gardnerella* exposures in naive mice. **Figure 1A** summarizes the experimental timeline. Our previous experiments demonstrated that *Gardnerella* is cleared from the mouse bladder within 12 h and that two exposures are required to elicit urothelial exfoliation (Gilbert et al., 1986). Presently, five female C57BL/6 mice were inoculated twice intravesically with *Gardnerella* strain JCP8151B. Four age-matched female C57BL/6 mice were inoculated twice with PBS in parallel (PBS) to serve as controls. Exposures were given 12 h apart, and bladders were collected 12 h after the second exposure.

**Figure 2:**
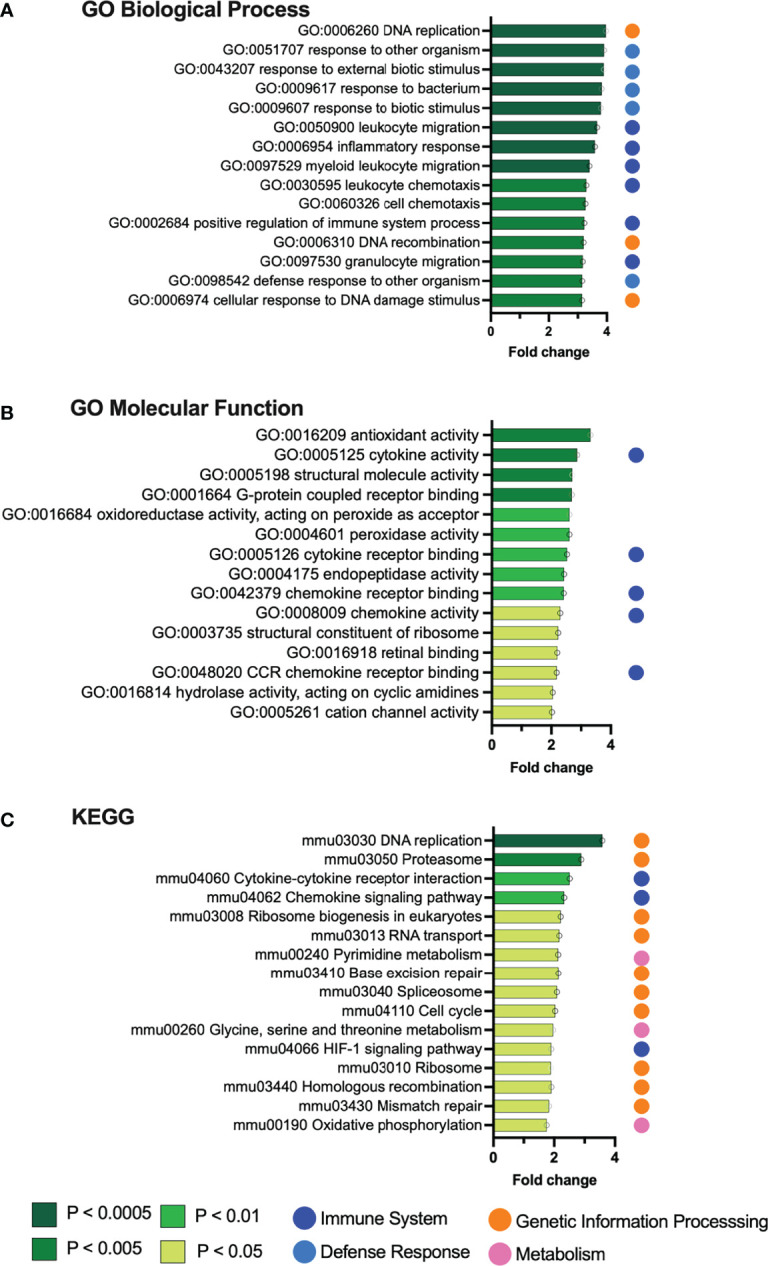
Gene set enrichment analysis identifies pathways upregulated by *Gardnerella* exposures. Graphs depict the top 15 gene sets from the Gene Ontology **(A, B)** and KEGG **(C)** databases that were significantly enriched in the bladders of mice exposed to *Gardnerella* relative to PBS controls.

RNA was extracted individually from each bladder and used for RNA-seq. A total of 305,893,605 RNA-seq reads were generated. Of these, 207,076,414 unique reads could be aligned to the *Mus musculus* reference genome. Further details of the RNA-seq reads are found in **Supplementary Table S1**. Differentially expressed genes (FDR adjusted *p* < 0.05, log_2_FC > 2) and pathways were identified by comparing bladders in the *Gardnerella* group to PBS controls. At the individual gene level, *Gardnerella* exposure resulted in significantly increased expression of 38 genes and decreased expression of 11 genes relative to PBS controls (**Figure 1B**; **Table 1**). Gene set enrichment analyses, using both the Gene Ontology (GO) and Kyoto Encyclopedia of Genes and Genomes (KEGG) pathway databases, were performed on the full RNA-seq dataset to gain a broader perspective of the biological effects of *Gardnerella* exposures on the bladder (**Figure 2**). The GO term analyses detected significant enrichment (*p* < 0.05, log_2_FC > 2) of 153 GO biological processes and 16 GO molecular functions (**Supplementary Tables S2**, **S3**) in *Gardnerella* bladders compared to PBS. Sixteen KEGG pathways were significantly enriched (*p* < 0.05, log_2_FC > 2) (**Supplementary Table S4**). There were very few significantly de-enriched pathways across all three categories; only the GO molecular function “pheromone activity” and the “steroid hormone biosynthesis” KEGG pathway were moderately decreased. In addition to single direction changes (all genes in the pathway either went UP or DOWN), seven KEGG pathways were significantly dysregulated in ANY direction (some genes in the same pathway went up and others went down) (**Supplementary Table S4**). As a whole, the RNA-seq data pointed to two broad categories affected by *Gardnerella* exposure: (1) inflammation and immune response and (2) urothelial exfoliation and differentiation.

**Table 1:** Genes differentially expressed in the bladder after two *Gardnerella* exposures.

Gene name	Description	logFC	*p*-value	FDR
Cxcl5	Chemokine (C-X-C motif) ligand 5 [Source : MGI Symbol;Acc : MGI:1096868]	8.11756	3.07E−07	4.98E−04
Trem1	Triggering receptor expressed on myeloid cells 1 [Source : MGI Symbol;Acc : MGI:1930005]	6.00981	1.85E−05	1.09E−02
Syngr4	Synaptogyrin 4 [Source : MGI Symbol;Acc : MGI:1928903]	5.43623	3.22E−05	1.54E−02
Clec4e	C-type lectin domain family 4, member e [Source: MGI Symbol;Acc : MGI:1861232]	5.23586	3.63E−07	5.64E−04
Krt6a	Keratin 6A [Source : MGI Symbol;Acc : MGI:1100845]	4.81622	2.32E−06	2.51E−03
1810065E05Rik	RIKEN cDNA 1810065E05 gene [Source : MGI Symbol;Acc : MGI:1917114]	4.72987	2.13E−05	1.19E−02
Ptpn5	Protein tyrosine phosphatase, non-receptor type 5 [Source : MGI Symbol;Acc : MGI:97807]	4.5585	1.09E−04	3.77E−02
Anxa10	Annexin A10 [Source : MGI Symbol;Acc : MGI:1347090]	4.14225	6.77E−08	1.42E−04
Bdkrb1	Bradykinin receptor, beta 1 [Source : MGI Symbol;Acc : MGI:88144]	4.03099	1.47E−07	2.76E−04
Chrnb4	Cholinergic receptor, nicotinic, beta polypeptide 4 [Source : MGI Symbol;Acc : MGI:87892]	3.95127	1.23E−04	4.11E−02
Fmo4	Flavin containing monooxygenase 4 [Source : MGI Symbol;Acc : MGI:2429497]	3.42047	4.32E−05	1.88E−02
Sprr2g	Small proline-rich protein 2G [Source : MGI Symbol;Acc : MGI:1330348]	3.40759	7.56E−07	1.04E−03
Fosl1	Fos-like antigen 1 [Source : MGI Symbol;Acc : MGI:107179]	3.27974	7.83E−07	1.04E−03
Vat1l	Vesicle amine transport protein 1 homolog-like (*T. californica*) [Source : MGI Symbol;Acc : MGI:2142534]	3.13659	1.53E−04	4.75E−02
Tff1	trefoil factor 1 [Source : MGI Symbol;Acc : MGI:88135]	3.1289	2.34E−05	1.25E−02
Ptx3	Pentraxin-related gene [Source : MGI Symbol;Acc : MGI:104641]	3.07996	2.16E−05	1.19E−02
Cml5	Camello-like 5 [Source : MGI Symbol;Acc : MGI:1916299]	3.06793	3.25E−06	3.23E−03
Cxcr2	Chemokine (C-X-C motif) receptor 2 [Source : MGI Symbol;Acc : MGI:105303]	3.02361	2.04E−06	2.35E−03
Pinlyp	Phospholipase A2 inhibitor and LY6/PLAUR domain containing [Source : MGI Symbol;Acc : MGI:3615324]	3.00226	5.61E−05	2.33E−02
Fam3b	Family with sequence similarity 3, member B [Source : MGI Symbol;Acc : MGI:1270150]	2.97956	1.23E−06	1.56E−03
Mefv	Mediterranean fever [Source : MGI Symbol;Acc : MGI:1859396]	2.97595	3.87E−05	1.75E−02
Gjb4	Gap junction protein, beta 4 [Source : MGI Symbol;Acc : MGI:95722]	2.93731	4.19E−05	1.85E−02
Csta1	Cystatin A1 [Source : MGI Symbol;Acc : MGI:3524930]	2.91392	6.98E−09	2.08E−05
Tnfaip6	Tumor necrosis factor alpha induced protein 6 [Source : MGI Symbol;Acc : MGI:1195266]	2.85446	2.18E−07	3.81E−04
Qrfpr	Pyroglutamylated RFamide peptide receptor [Source : MGI Symbol;Acc : MGI:2677633]	2.84716	1.08E−05	6.92E−03
Gm10309	Predicted gene 10309 [Source : MGI Symbol;Acc : MGI:3641941]	2.82107	1.25E−04	4.12E−02
Rnf183	Ring finger protein 183 [Source : MGI Symbol;Acc : MGI:1923322]	2.76461	8.21E−06	5.98E−03
Krt13	Keratin 13 [Source : MGI Symbol;Acc : MGI:101925]	2.64816	6.48E−05	2.63E−02
Serpina3m	Serine (or cysteine) peptidase inhibitor, clade A, member 3M [Source : MGI Symbol;Acc : MGI:98378]	2.53427	3.16E−06	3.23E−03
Mmp10	Matrix metallopeptidase 10 [Source : MGI Symbol;Acc : MGI:97007]	2.5287	1.30E−04	4.21E−02
Ch25h	Cholesterol 25-hydroxylase [Source : MGI Symbol;Acc : MGI:1333869]	2.49894	1.77E−06	2.15E−03
Socs3	Suppressor of cytokine signaling 3 [Source : MGI Symbol;Acc : MGI:1201791]	2.46996	1.02E−04	3.57E−02
Usp2	Ubiquitin specific peptidase 2 [Source : MGI Symbol;Acc : MGI:1858178]	2.41504	4.93E−10	2.94E−06
Timp1	Tissue inhibitor of metalloproteinase 1 [Source : MGI Symbol;Acc : MGI:98752]	2.22733	5.90E−06	4.79E−03
Lonrf3	LON peptidase N-terminal domain and ring finger 3 [Source : MGI Symbol;Acc : MGI:1921615]	2.20086	1.56E−05	9.61E−03
Nts	neurotensin [Source : MGI Symbol;Acc : MGI:1328351]	2.10926	1.30E−04	4.21E−02
Nr4a2	Nuclear receptor subfamily 4, group A, member 2 [Source : MGI Symbol;Acc : MGI:1352456]	2.10351	3.39E−08	8.08E−05
Errfi1	ERBB receptor feedback inhibitor 1 [Source : MGI Symbol;Acc : MGI:1921405]	2.05576	3.84E−09	1.25E−05
Nr1d1	Nuclear receptor subfamily 1, group D, member 1 [Source : MGI Symbol;Acc : MGI:2444210]	−2.14917	1.41E−13	2.52E−09
2310015D24Rik	RIKEN cDNA 2310015D24 gene [Source : MGI Symbol;Acc : MGI:1917350]	−2.18627	9.74E−05	3.50E−02
Egr1	early growth response 1 [Source : MGI Symbol;Acc : MGI:95295]	−2.28215	2.16E−09	7.72E−06
Snora31	Small nucleolar RNA, H/ACA box 31 [Source : MGI Symbol;Acc : MGI:3819500]	−2.31741	6.27E−06	4.98E−03
Gm15883	Predicted gene 15883 [Source : MGI Symbol;Acc : MGI:3801875]	−2.73333	1.61E−04	4.95E−02
Gm12426	Predicted gene 12426 [Source : MGI Symbol;Acc : MGI:3650989]	−3.28567	9.06E−05	3.41E−02
Fos	FBJ osteosarcoma oncogene [Source : MGI Symbol;Acc : MGI:95574]	−3.8295	2.01E−17	7.18E−13
1200007C13Rik	RIKEN cDNA 1200007C13 gene [Source : MGI Symbol;Acc : MGI:1921369]	−3.85771	8.66E−05	3.37E−02
Gm26887	Predicted gene, 26887 [Source : MGI Symbol;Acc : MGI:5477381]	−4.04586	1.78E−05	1.06E−02
Gm5828	Predicted gene 5828 [Source : MGI Symbol;Acc : MGI:3644176]	−4.35603	8.69E−05	3.37E−02
Igkv12-46	Immunoglobulin kappa variable 12-46 [Source : MGI Symbol;Acc : MGI:4439773]	−7.10718	3.20E−06	3.23E−03

### Inflammatory Pathways Are Upregulated After *Gardnerella* Exposures

As would be expected in a bacterial exposure model, most of the gene sets enriched in *Gardnerella*-exposed bladders were related to inflammatory responses. We previously reported higher levels of bladder IL-12p40 following *Gardnerella* exposure (Gilbert et al., 2017). The most highly upregulated GO biological processes (**Figure 2A** **)** were directly related to inflammation, including leukocyte migration and chemotaxis (dark blue dots) or the more general “response to…” terms such as “bacterium” or “external biotic stimulus” (light blue dots). This theme continued through the rest of the enriched GO biological process terms; 49 were related to immune and inflammatory processes involving cytokines, chemokines, and leukocytes (**Supplementary Table S2**), and 15 were relevant “response to…” and “defense response to…” terms such as “bacterium,” “molecule of bacterial origin,” “external biotic stimulus,” and “other organism” (**Supplementary Table S2**). Activation of host inflammatory responses was also reflected in the upregulated GO molecular functions, with top hits indicating cytokine and chemokine activity and chemokine receptor binding (**Figure 2B**). Likewise, “cytokine–cytokine receptor interactions” and “chemokine signaling pathway” were among the upregulated KEGG pathways, as well as the HIF-1α signaling pathway that is known to mediate the host inflammatory response to bacteria (**Figure 2C**). Among the individual upregulated genes (**Table 1**) were the inflammatory mediator *Cxcl5* and its cognate receptor *Cxcr2*, the antimicrobial peptide *Ptx3*, the innate immune cell activating lectin *Clec4e* (also known as Mincle), and *Trem1*, which is expressed on myeloid cells and stimulates release of inflammatory cytokines in response to pathogens (Tessarz and Cerwenka, 2008). Given the gene expression signature of inflammation, we examined whether *Gardnerella-*exposed bladders collected at the same time point used for RNA-seq analysis displayed robust neutrophil migration into the urothelium like what has been seen during bladder infection with established uropathogens (Mulvey et al., 2000; Mulvey et al., 2001). However, we did not observe robust neutrophil infiltration into the urothelium in any of the *Gardnerella*-exposed bladders (**Supplementary Figure S1**), suggesting that either more time or additional exposures may be required to effect changes at the level of neutrophil recruitment, or that a different cellular response occurs. Future time-course experiments and more in-depth histological analysis by a pathologist and assessment of specific immune cell populations and activation states in the bladder by flow cytometry are needed to distinguish these possibilities.

### Upregulated Genes and Pathways Reflect *Gardnerella*-Induced Urothelial Exfoliation

**Figure 3:**
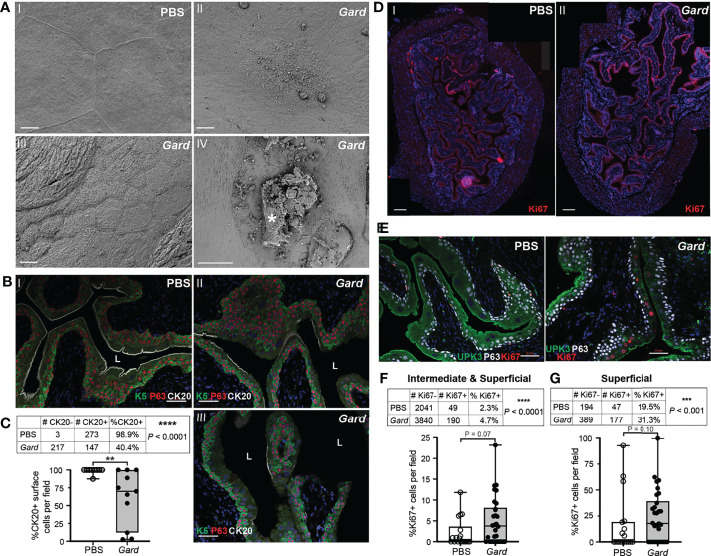
*Gardnerella* exposures result in urothelial exfoliation and proliferation. **(A)** Scanning electron micrographs of bladders collected 12 h after two exposures (the model used for RNA-seq, see **Figure 1** schematic) to PBS (panel I) or *Gardnerella* (II–IV). (I) Intact superficial umbrella cells; (II) umbrella cell with membrane blebbing; (III) region of exfoliation with smaller underlying intermediate cells visible at the luminal interface; (IV) exfoliating umbrella cell marked by an asterisk. Scale bars = 10 μm. **(B)** Immunofluorescence microscopy of urothelial differentiation markers in bladder sections from mice exposed to PBS (I) or *Gardnerella* (II and III). Keratin 5 (K5) in green labels, basal cells; P63 in red labels, basal and intermediate cells; and cytokeratin 20 (CK20) in white labels, the apical surface of fully differentiated umbrella cells. Scale bars = 50 μm. **(C)** Counts of CK20-positive (+) and CK20-negative (−) cells on the urothelial surface, adjacent to the lumen. ^****^ *p* < 0.001, Fisher’s exact test. The graph was generated by plotting the %CK20 positivity, with each dot representing an individual microscopy image of the ×20 field of view. ^**^ *p* < 0.01, Mann–Whitney *U* test. Box plot denotes the 25th and 75th percentiles with a line at the median and whiskers from min to max. **(D)** Panoramic assembly of bladder sections stained for Ki67 in red. Scale bars = 200 μm. **(E)** Representative images of immunofluorescence microscopy used for Ki67+ enumeration. **(F, G)** Tables show the counts of Ki67-positive (+) and Ki67-negative (−) cells of superficial and intermediate cells combined **(F)** or only superficial cells **(G)** in each experimental group. ^***^ *p* < 0.001, ^****^ *p* < 0.0001, Fisher’s exact test. Graphs were generated by plotting the %Ki67 positivity, with each dot representing an individual microscopy image as in **(B)**. Box plot denotes the 25th and 75th percentiles with a line at the median and whiskers from min to max.

Several significantly upregulated gene sets were related to urothelial integrity and turnover. ‘DNA replication’ was the top hit in both the KEGG and GO biological process lists (**Figure 2**). Additional terms indicating cell proliferation in the *Gardnerella*-exposed bladders were those related to cell cycle, ribosome biogenesis, translation, cell activation, and nuclear division (**Supplementary Tables S2** **–** **S4** **)**. The “neuroactive ligand–receptor interaction,” “cytokine–cytokine receptor interaction,” and “retinol metabolism” (Lu et al., 2021) KEGG pathways have been linked to bladder cancer (Zhang et al., 2021). Several of the individual genes upregulated by *Gardnerella* exposures are involved in epithelial to mesenchymal transition (*Cxcl5*, *Cxcr2*, *Tff1*) (Lee et al., 2021) or known to be elevated during squamous metaplasia or bladder cancer (*Anxa10*, *Fosl1*, *Krt6a*, *Mmp10*) (Cao et al., 2010; Somji et al., 2011; Gatta et al., 2019; Kudelski et al., 2021; Wu et al., 2021). These RNA-seq data are consistent with our previous findings, reproduced here, that two *Gardnerella* exposures trigger membrane blebbing and exfoliation of superficial umbrella cells lining the bladder lumen (**Figure 3A**) (Gilbert et al., 2017). To further corroborate the RNA-seq signature of urothelial turnover, additional bladders were examined for markers of urothelial differentiation and proliferation. Compared to bladders exposed only to PBS, three out of four bladders exposed to *Gardnerella* displayed a noticeable increase in urothelial keratin 6 (Krt6), a marker of squamous differentiation, which is consistent with the increased *Krt6* transcript detected by RNA-seq (**Supplementary Figure S2**). Another marker of urothelial differentiation is cytokeratin 20: its appearance on the apical membrane marks the last event in differentiation of superficial umbrella cells (Veranic et al., 2004). Bladders from mice exposed only to PBS had cytokeratin 20 (CK20) staining that was contiguous across the length of the urothelial-luminal interface, indicating an intact and fully differentiated urothelium (**Figure 3B** (I). In contrast, the urothelium from mice exposed to *Gardnerella* had regions lacking CK20 staining (**Figure 3B** (II and III). We enumerated CK20-positive superficial cells lining the urothelial surface. We also counted the total number of superficial cells (those at the luminal interface) in order to determine the percentage that were CK20 positive. These data confirmed a significant decrease in CK20 staining in mice exposed to *Gardnerella* compared to PBS controls (**Figure 3C**). The increased Krt6 and decreased CK20 staining pattern observed on *Gardnerella*-exposed bladders suggested that exfoliation had occurred in these areas and the urothelium had not completely healed. Finally, we examined cell proliferation *via* Ki67 staining. Naive, unperturbed adult mouse bladders have a very slow urothelial turnover and thus contain few to no Ki67-positive cells. Exfoliation of superficial urothelial cells is known to trigger proliferation and differentiation of underlying cells to restore the urothelium. In our model, Ki67-positive cells were present in *Gardnerella*-exposed bladders (**Figure 3D** (II), but they were also seen in bladders exposed to PBS, reflecting the fact that the transurethral inoculation procedure itself perturbs the bladder tissue (**Figure 3D** (I). To determine whether there was an overall increase in proliferation in *Gardnerella-*exposed mice compared to PBS controls, we enumerated Ki67-positive cells in the transitional urothelium (**Figures 3E–G**). We also counted the total number of cells in order to determine the percentage that were Ki67 positive. When the transitional urothelium was analyzed (intermediate and superficial cells), the proportion of Ki67-positive cells was significantly higher in mice exposed to *Gardnerella* than in PBS controls (**Figure 3F**). This increase in Ki67 positivity in *Gardnerella-*exposed bladders was apparent even if only superficial cells were analyzed (**Figure 3G**). Our previous data suggested that exfoliating cells are dying *via* apoptosis since *Gardnerella-*exposed bladders exhibited increased cleaved Casp-3 staining and TUNEL-positive urothelial cells (Gilbert et al.). Consistent with this phenotypic data, *Gardnerella* exposures increased 15 GO terms related to apoptosis (**Supplementary Table S2**). These data, together with our previous findings, demonstrate that bladder exposures to *Gardnerella* result in urothelial exfoliation.

### *Gardnerella* Preexposures Promote Acute UPEC Bacteriuria

**Figure 4:**
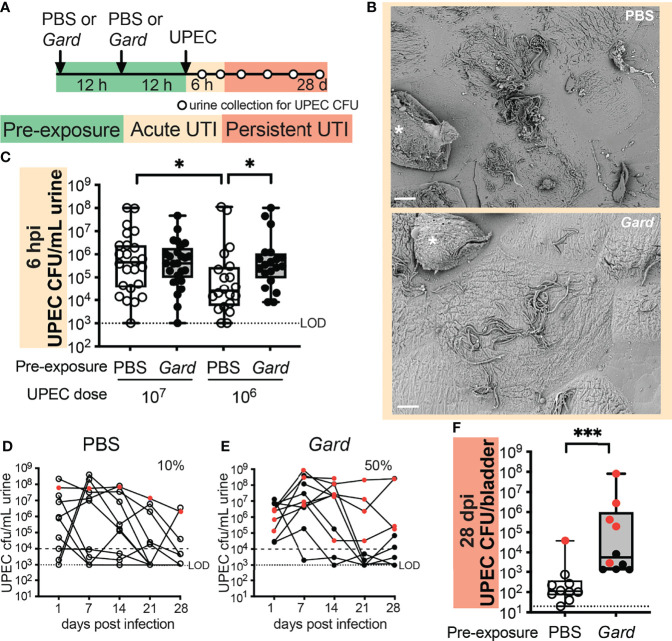
Preexposure to *Gardnerella* increases acute UPEC bacteriuria and persistent infection. **(A)** Schematic of the mouse experiment time course. Dots on the timeline indicate when urine was collected for CFU enumeration. Data are from five independent mouse experiments. **(B)** SEM images of bladders collected 6 h after UPEC infection from mice preexposed to PBS (top) or *Gardnerella* (bottom). Asterisks mark exfoliating umbrella cells. Scale bars = 20 μm. Refer to **Supplementary Figure S3** for additional bladder SEM images. **(C)** Acute UPEC titers in urine collected 6 hpi, with each dot representing an individual mouse (10^7^ *n* = 20 per group; 10^6^ PBS *n* = 24, *Gardnerella n* = 25). ^*^ *p* < 0.05, Mann–Whitney *U* test. Box plot denotes the 25th and 75th percentiles with a line at the median and whiskers from min to max. **(D, E)** Time course of UPEC bacteriuria after a 10^7^ CFU inoculation. Red symbols indicate mice that had UPEC bacteriuria >10^4^ CFU/ml in each weekly urine sample. **(F)** UPEC titers in bladder homogenates collected 28 dpi. Each dot represents an individual mouse (*n* = 10 per group). Red symbols indicate mice that had UPEC bacteriuria >10^4^ CFU/ml in each weekly urine sample. LOD, limit of detection. ^***^ *p* < 0.001, Mann–Whitney *U* test. Box plot denotes the 25th and 75th percentiles with a line at the median and whiskers from min to max.

Urothelial turnover and inflammatory responses in the bladder are important features of UTI caused by established uropathogens such as UPEC (Lacerda Mariano and Ingersoll, 2020). Given our RNA-seq results, we hypothesized that *Gardnerella* exposure would affect the course of an experimental UPEC UTI. To examine this hypothesis, we developed a “preexposure” model in which mice received *Gardnerella* exposures before UPEC inoculation (**Figure 4A**). We exposed mice twice, 12 h apart, to *Gardnerella* as in the RNA-seq experiment. Twelve hours after the second *Gardnerella* or PBS control preexposure, mice were inoculated transurethrally with the UPEC clinical isolate UTI89. The UTI89 strain is widely used in experimental UTI models, including in our prior study that reported that *Gardnerella* exposures induced recurrent UPEC UTI from intraepithelial reservoirs (Gilbert et al., 1986).

The UPEC pathogenic cascade involves urothelial attachment, invasion, and intracellular replication followed by filamentation and egress (Klein and Hultgren, 2020). We hypothesized that the urothelial exfoliation resulting from *Gardnerella* exposure could disrupt or alter the interaction of UPEC with the urothelium. First, we directly examined UPEC-bladder interactions in our preexposure model using scanning electron microscopy (SEM). Consistent with previous reports in naive mice, UPEC-infected mice preexposed to PBS harbored abundant UPEC adhering to, and filamenting out of, superficial urothelial cells as well as exfoliating cells full of intracellular bacteria (**Figure 4B**, top). Similar features were also seen on the superficial cells of bladders preexposed to *Gardnerella* (**Figure 4B**, bottom), suggesting that the urothelium was still able to harbor intracellular UPEC. One noticeable difference was that some of the superficial cells in bladders preexposed to *Gardnerella* had small collections of adherent bacillary UPEC in addition to the filamentous bacteria more typically observed in control bladders (**Supplementary Figure S3**, arrowheads). Ultimately, the SEM analysis demonstrated that preexposure to *Gardnerella* did not entirely disrupt the UPEC invasion and egress cycle.

Next, we took a more quantitative approach by examining UPEC titers in urine in our preexposure model. With an inoculum of 10^7^ colony-forming units (CFUs) of UPEC, which is frequently used in mouse UTI models (Conover et al., 2015;Gilbert et al.), UPEC urine titers were indistinguishable between PBS preexposed and *Gardnerella* preexposed mice at 6 and 24 hpi (**Figure 4C**; **Supplementary Figure S4**). We reasoned that the effects of *Gardnerella* might be masked in the context of the high titer UPEC inoculum, especially at early time points. Therefore, we also examined a lower dose of 10^6^ CFUs of UPEC. Strikingly, UPEC bacteriuria was significantly higher at 6 hpi in mice preexposed to *Gardnerella* than in PBS preexposed controls (**Figure 4C**). These data suggest that alterations in the bladder niche caused by preexposures to *Gardnerella* promote UPEC bacteriuria.

### *Gardnerella* Exposures Promote UPEC Persistence in the Bladder

Since we observed an effect of *Gardnerella* preexposures on acute bacteriuria, we examined whether this would extend to later time points and whether the effect would be observable in bladder tissue in addition to urine. Wild-type C57BL/6 female mice are generally not susceptible to persistent (chronic) bacteriuria following a single UPEC inoculation (Schwartz et al., 2015). Consistent with prior reports, most of the mice inoculated with 10^6^ CFUs of UPEC cleared bacteriuria by 24 hpi, with no apparent difference between preexposure groups (**Supplementary Figure S4**). With an inoculum of 10^7^ CFUs of UPEC, only 10% of mice in the PBS control preexposure group had UPEC bacteriuria that persisted at >10^4^ CFU/ml out to 4 weeks postinfection (wpi) (**Figure 4D**), compared with 50% in mice preexposed to *Gardnerella* (**Figure 4E**). Previous studies have shown that mice that develop persistent bacteriuria have higher levels of the proinflammatory cytokines IL-5, IL-6, KC, and G-CSF in serum at 24 hpi compared to those that ultimately clear the infection (Hannan et al., 2010; Schwartz et al., 2015). We hypothesized that *Gardnerella* exposure alone may result in a similar cytokine signature, and if this were to occur prior to UPEC introduction into the bladder, it could prime the host for the development of persistent UPEC infection. To test this hypothesis, we measured cytokine levels in bladders that were exposed twice to *Gardnerella* and were collected at the time point that they would have received UPEC in our preexposure model. However, inconsistent with our hypothesis, there was no difference in serum levels of IL-5, IL-6, KC, or G-CSF (**Supplementary Figure S5**) or in any of the other 23 cytokines/chemokines measured (data not shown) in mice preexposed to *Gardnerella* versus to PBS.

Bladder titers of UPEC at 4 wpi were significantly higher in mice exposed to *Gardnerella* compared to PBS controls (*p* = 0.0003; **Figure 4F**). This difference remained significant even when the bladders from mice without persistent UPEC bacteriuria were analyzed separately (**Supplementary Figure S6**). This is notable because UPEC that is detectable in bladder tissues in the absence of bacteriuria has previously been shown to reside in quiescent intracellular reservoirs (Mulvey et al., 2001), suggesting that preexposure to *Gardnerella* may promote the formation of more, or larger, reservoirs. Future studies using methods that distinguish intracellular from luminal UPEC are needed to distinguish these possibilities. Taken together, these initial studies demonstrate that preexposure to *Gardnerella* promotes both acute and persistent UPEC infection in the bladder.

## Discussion

*Gardnerella* is a common, and often dominant, member of the vaginal microbiome, especially in the context of dysbiosis (Morrill et al., 2020). Likewise, *Gardnerella* has recently been recognized as a frequent, dominant member of the urobiome, including in studies that used collection methods to limit contamination by periurethral or vaginal organisms (Birch et al., 1981; McDowall et al., 1981; McDonald et al., 1982; Fairley and Birch, 1983; Gilbert et al., 1986). Despite *Gardnerella* being frequently isolated from urine, relatively little is known regarding the biological effects of *Gardnerella* on bladder tissue. To address this lack of knowledge, we recently developed a model of *Gardnerella* bladder exposure in female mice (O'Brien et al., 2020). Here, we further characterized our model using whole bladder RNA-seq to determine the bladder transcriptomic response to *Gardnerella*. The RNA-seq data, along with additional microscopy validation studies, echo our previous report of *Gardnerella*-induced urothelial apoptosis and exfoliation (Gilbert et al., 2017). The RNA-seq results further revealed that *Gardnerella* activates host transcriptional pathways related to mucosal inflammation and immunity. Taken together, these data provide evidence that bacterial species such as *Gardnerella*, that have been frequently identified in urinary microbiome studies but have remained understudied, have observable biological effects on the bladder tissue in a relevant *in vivo* model.

To further explore the potential clinical relevance of *Gardnerella* bladder exposures, here, we focused on the effect of *Gardnerella* on UTIs caused by UPEC. We chose to focus first on UPEC UTI for the following reasons: First, women with BV, who thus have high levels of *Gardnerella*, are at increased risk of UTI (Hooton et al., 1989; Sumati and Saritha, 2009; Hillebrand et al., 2022). Second, sexual activity, which likely results in bladder exposure to urogenital bacteria like *Gardnerella*, is one of the strongest risk factors for UTI (Nicolle et al., 1982; Foxman, 2014). Of particular relevance to these first two points, one study found that the strongest correlation between the vaginal and urinary microbiome occurred in women with BV (Gottschick et al.). Third, modulation of the urobiome has been proposed as an antibiotic-sparing alternative therapeutic approach to treat or prevent UTIs (Jung and Brubaker, 2019; Cole et al., 2021; Jones et al., 2021; Garofalo et al., 2022), but the direct impact of urobiome members on UTI outcomes have not been examined *in vivo*. Fourth, the most notable effects of *Gardnerella* in our RNA-seq dataset were urothelial integrity and inflammation, which are known to be key host determinants of UPEC UTI. In summary, the convergence of data from clinical studies and our *Gardnerella* bladder exposure model led us to extend our model to examine the effects of *Gardnerella* exposure on outcomes of UTIs caused by UPEC. Data from this model provide evidence that preexposures to *Gardnerella* can enhance UPEC acute and persistent UTIs. *Gardnerella* preexposures enhanced UPEC bacteriuria, at an early time point, in a manner dependent on UPEC dose. The effect of *Gardnerella* on acute UPEC bacteriuria was only evident when mice were given a relatively lower UPEC inoculum. This observation suggests that *Gardnerella* bladder exposures could lower the threshold dose required for UPEC to establish UTI in women. Additionally, preexposure to *Gardnerella* rendered mice more susceptible to persistent UPEC bacteriuria and increased UPEC burden in bladder tissue. The increase in bladder UPEC was detected even in mice without persistent UPEC bacteriuria. The presence of UPEC in bladder tissue in the absence of bacteriuria has previously been attributed to stable UPEC reservoirs within urothelial cells that can later emerge to cause a recurrent UTI (Mulvey et al.). The data presented here warrant future studies using established assays (e.g., immunofluorescence microscopy, gentamicin protection) to examine UPEC intracellular niche distribution in the context of *Gardnerella* exposure. Taken together, these data demonstrate that bladder exposures to *Gardnerella* enhance UPEC UTI in a relevant *in vivo* model and provide further biological explanation for the association between BV and UTI observed in women.

While it is evident from the data presented here that *Gardnerella* exposures enhance UPEC UTI, the molecular mechanisms driving this effect remain to be determined. Despite *Gardnerella* activating transcription of inflammatory pathways, we observed no evidence of tissue inflammation. However, it remains possible that distinct immune cell populations are responding to *Gardnerella* in ways that require more focused assays to detect. So far, the data point to the effect of *Gardnerella* on the urothelium as the most likely source of influence on UPEC UTI. Future experiments could test this idea by attempting to block exfoliation using cell-death pathway inhibitors. Prior studies have pointed to host responses happening during the acute stages of UPEC infection as drivers of chronic outcomes (Hannan et al., 2010; Schwartz et al., 2015). We expect that a similar situation is occurring in our preexposure model, meaning that whatever is promoting UPEC persistence in *Gardnerella*-exposed mice occurs during the early stages of UPEC infection. We do not expect that *Gardnerella* directly impacts the bladder tissue 1 month after exposure because *Gardnerella* is cleared from the mouse urinary tract by 12 h (Gilbert et al.).

A limitation of our study was that we only examined a single time point. Also, since we examined bladders 12 h after two exposures to *Gardnerella*, we cannot distinguish whether the differences required two exposures or if they would have occurred 24 h following one exposure. Since whole bladders were analyzed, we cannot attribute transcriptional changes to specific regions or cell types of the bladder, and RNA-seq analysis will not uncover epigenetic changes that have occurred. Another limitation was that we only examined one strain of *Gardnerella* (JCP8151B) that is a vaginal isolate and which may become reclassified as a species other than *vaginalis* (but was regarded as *G. vaginalis* when we performed our study). Studies examining associations between *Gardnerella* in the urobiome and various conditions associated with lower urinary tract symptoms, such as urgency urinary incontinence and overactive bladder, have yielded mixed results. However, many of these studies only examined the urobiome at the genus level and did not distinguish between *Gardnerella* species or subgroups. It is possible that the inconsistency in associations with lower urinary tract symptoms reflects that different *Gardnerella* species have greater or less capacity to influence the bladder. It has been noted that *G. piotii* has not yet been isolated from urine, while most other species and subspecies of *Gardnerella* have been isolated from both niches (Putonti et al., 2021). However, this could be attributed to differences in the culture techniques that were used in studies isolating *Gardnerella* from the urine compared to those that isolated *Gardnerella* from the vagina. Whether or not different *Gardnerella* species or subgroups display different colonization kinetics or pathologic features in the bladder can be directly tested in mice using our *Gardnerella* exposure model.

In addition to the connection to UTI, the genes and biological pathways affected by *Gardnerella* in our mouse model are related to bladder function and intersect with a wide range of other urological conditions, including but not limited to bladder cancer, urinary incontinence, bladder pain syndrome and interstitial cystitis. For example, cholinergic and bradykinin receptors and the neuropeptide neruotensin (Nts) mediate bladder muscle cell contraction (Dong et al., 2015; Dalghi et al., 2020; Borsodi et al., 2021). Type 1 bradykinin receptors like Bdkrb1 are generally not expressed in healthy tissue but are induced by inflammatory mediators and injury (Marceau et al., 1997), which is consistent with the increase in expression after *Gardnerella* exposure. The nicotinic cholinergic receptor that was induced by *Gardnerella*, Chrnb4, is expressed by bladder afferent neurons and is necessary for strips of bladder tissue to contract in response to nicotine stimulation *ex vivo* (Xu et al., 1999). The Human Phenotype Ontology database reports that the orphan nuclear receptor Nr4a2 is associated with bladder function and urinary urgency. Dysregulation of the “neuroactive ligand–receptor interaction” and “phototransduction” KEGG pathways further suggests an influence of *Gardnerella* exposures on the bladder–brain axis. The observation that *Gardnerella* exposure induced genes related to bladder sensation and urination is noteworthy because some studies in women have detected an association between the presence of *Gardnerella* in urine and urgency urinary incontinence. Future studies could expand the exposure model to investigate the effect of *Gardnerella* on urination frequency and other measures of bladder function.

In summary, here, we demonstrate that *Gardnerella* directly impacts the bladder, activating transcriptional inflammatory responses and causing urothelial exfoliation and turnover. We present further evidence of *Gardnerella* as a “covert pathogen” in the bladder (Gilbert and Lewis, 2019), affecting outcomes of UPEC UTI at time points long after *Gardnerella* has been cleared from the bladder. These findings have important implications for how we think about the potential influence of urobiome bacteria on disease outcomes in the bladder.

## Materials and Methods

### Ethics Statement

Mouse experiments were carried out in strict accordance with the recommendations in the Guide for the Care and Use of Laboratory Animals. The Institutional Animal Care and Use Committee (IACUC) of Washington University School of Medicine approved all procedures in advance (Protocol Numbers: 20170081 and 20-0031).

### Bacterial Strains and Growth Conditions

*Gardnerella* strain JCP8151B (Lewis et al., 2013) was grown anaerobically at 37°C in static liquid culture in NYCIII medium for 16 h or on NYCIII agar plates with 1 mg/ml streptomycin. Uropathogenic *E. coli* strain UTI89, harboring a kanamycin resistance cassette (Wright et al., 2005), was grown aerobically at 37°C in static liquid culture in Lysogeny Broth (LB) medium for 18 h and subcultured 1:1,000 in fresh LB for 18 h or on LB agar plates with 25 mg/ml kanamycin. Mouse inocula were prepared as previously described (O'Brien et al., 2020).

### Mice

Six- to seven-week-old female C57BL/6 mice were obtained from Charles River (Fredericks facility). Mice were given a regular chow diet in a specific pathogen-free facility with a 12-h light/12-h dark cycle at Washington University School of Medicine. Mice were allowed to acclimate to the facility after transport for 1 week prior to experiments.

### Mouse Urinary Tract Inoculation Experiments for RNA-seq

Experiments were performed essentially as described previously (O'Brien et al., 2020). Briefly, mice were anesthetized with isoflurane and then inoculated transurethrally with 50 μl prepared inoculum of 1 × 10^8^ CFU *Gardnerella* strain JCP8151B (5 mice) or PBS (4 mice). Twelve hours later, mice received a second transurethral inoculation of *Gardnerella* or PBS. Twelve hours later, all mice were humanely sacrificed by cervical dislocation under isoflurane anesthesia, and bladders were aseptically harvested and flash frozen in liquid nitrogen for future RNA isolation.

### Library Preparation and Sequencing

Bladders were homogenized and RNA was extracted using the RNeasy Plus Mini kit (Qiagen). Libraries were prepared from each bladder individually with 10 ng of total RNA, and RNA integrity was determined using an Agilent Bioanalyzer, with a Bioanalyzer RIN score >8.0 obtained for all samples. ds-cDNA was prepared using the SMARTer Ultra Low RNA Kit for Illumina Sequencing (Takara-Clontech) per the manufacturer’s protocol. cDNA was fragmented using a Covaris E220 sonicator using peak incident power of 18, duty factor 20%, cycles/burst 50, time 120 s to yield an average size of 200 base pairs (bp). cDNA was then blunt ended, had an A base added to the 3′ ends, and then had Illumina sequencing adapters ligated to the ends. Ligated fragments were then amplified for 12 cycles using primers incorporating unique index tags. Fragments were multiplexed with 5–6 samples per lane and were sequenced on an Illumina HiSeq 2500 using single-end 50 bp reads to target 30 M reads per sample.

### RNA-seq Data Acquisition, Quality Control, and Processing

RNA-seq reads from the nine individual libraries were demultiplexed using a custom demultiplexing script written in Python and then aligned to the Ensembl GRCm38.76 (*Mus musculus*) assembly with STAR version 2.0.4b. Subread:featureCount version 1.4.5 was used to derive gene counts from the number of uniquely aligned unambiguous reads. Sailfish version 0.6.3 was used to produce transcript counts. RSeQC version 2.3 was used to assess sequencing performance for total number of aligned reads, total number of uniquely aligned reads, genes and transcripts detected, ribosomal fraction, known junction saturation, and read distribution over known gene models. All gene-level and transcript counts were then imported into the R/Bioconductor package EdgeR and TMM-normalized to adjust for differences in library size. Genes or transcripts not expressed in any sample were excluded from further analysis. Spearman correlation matrix and multidimensional scaling plots were used to assess the performance of the samples. Generalized linear models with robust dispersion estimates were created to test for gene/transcript level differential expression. The fits of the trended and tagwise dispersion estimates were then plotted to confirm proper fit of the observed mean to variance relationship where the tagwise dispersions are equivalent to the biological coefficients of variation of each gene. Differentially expressed genes and transcripts (comparing PBS vs. *Gardnerella*) were then filtered for FDR-adjusted *p*-values less than or equal to 0.05. Global perturbations in known GO terms and KEGG pathways were detected for each EdgeR contrast using the R/Bioconductor package GAGE to test for changes in expression of the reported log_2_ fold-changes reported by edgeR in each term versus the background log_2_ fold-changes of all genes found outside the respective term.

### Deparaffinization and Antigen Retrieval

Additional bladders were collected and fixed overnight in 4% paraformaldehyde at 4°C with gentle shaking and then transferred to 70% ethanol. Bladders were embedded in paraffin and sagittal sections were prepared and mounted on glass slides. Slides were placed onto glass holding trays and then placed into fresh Histo-Clear® Histological Clearing Agent, National Diagnostics, for 10 min two times. The trays were drained, then moved to 100% ethyl alcohol for 10 min two times, and then to 95% ethyl alcohol two times for 10 min. Finally, glass trays holding the slides were placed under running water for 10 min. During the deparaffinization, fresh pH 9 and 6 buffered antigen retrieval solutions were made and brought to a boil in 50 ml BD conical tubes in a glass beaker filled with water in a steamer. After washing, glass slides were placed into the appropriate buffer around 90°C–100°C without allowing the slides to dry out and boiled for 15 and 30 min in for pH 9 and pH 6 buffer, respectively. Slides were then cooled and allowed to cool to 60°C and were washed in 0.5% Triton X-100 in phosphate-buffered saline (PBS) at room temperature.

### Antibody and Histology Staining

For histological analysis, slides were stained with hematoxylin and eosin (H&E) according to standard protocol. For immunostaining, glass slides were removed from wash buffer one at a time, placed horizontally into humidified slide boxes, and the hydrophobic boundary was marked around the perimeter with a PAP PEN. About 300 μl of 10% heat-inactivated horse serum (HIHS) and 3% bovine serum albumin (BSA) in 0.5% Triton X-100 PBS were placed onto each slide for blocking. Slides were incubated in a closed, humidified slide box for 1 to 2 h. During blocking, primary antibody cocktails of chicken anti-KRT 5 1:500; goat anti-P63 1:300; mouse anti-cytokeratin 20 1:200 or rabbit anti-Krt6a 1:1,500; rabbit anti-Ki67 1:200; and mouse anti-Upk3 1:50 were prepared in 1% HIHS and 1% BSA in 0.5% Triton X-100 sufficient for around 300 μl per slide. The blocking solution was removed by vacuum and the primary antibody added onto the slide without disturbing hydrophobic perimeter. Slides were incubated overnight in humidified slide boxes at 4°C.

The following day, the primary antibody was removed, and the slides were washed for 10 min in fresh 0.5% Triton X-100 PBS twice. During washes, secondary antibody cocktails were prepared in1% HIHS and 1% BSA in 0.5% Triton X-100 for 300 μl per slide, and two drops of NucBlue® nuclear staining reagent (DAPI) were added per milliliter of antibody cocktail. Slides were removed from the washing buffer and hydrophobic perimeters redrawn then a secondary antibody cocktail was added. Slides were incubated in the dark at room temperature for 30 min to 1 h. After incubation, slides were washed, and previously warmed DAKO glycerol mounting medium was applied before the coverslip. Slides were stored overnight at 4C in slide folders.

### Immunofluorescent and Brightfield Imaging and Analysis

Fluorescent images were acquired with a Zeiss Axiovert 200M microscope with Zeiss Apotome as previously described (Tate et al., 2021). Urothelium cell types were distinguished and counted as: basal cells Krt5+P63+, intermediate cells P63+Krt5−, and superficial cells were P63−CK20+. The analysis of CK20 staining used 2 mice per experimental group, with three nonadjacent sections counted from each bladder. Cells at the luminal interface were enumerated as CK20+ or CK20− in 1–2 images from each bladder section (>275 total cells counted per group). The analysis of Ki67 staining used 3 mice per experimental group, with two nonadjacent sections counted from each bladder. Superficial (C20+P63−) and intermediate cells (P63+Krt5−) were enumerated as Ki67+ or Ki67− in 3–5 images from each bladder section (>2,000 total cells counted per group). Bright-field images were collected using a Nikon Eclipse TE200 microscope. Data were analyzed using the Fiji package of ImageJ.

### Scanning Electron Microscopy

Bladders were fixed *in situ* with EM fixative (2% paraformaldehyde, 2% glutaraldehyde in 0.1 M sodium phosphate buffer, pH 7.4) as previously described (O'Brien et al., 2020). Samples were postfixed in 1.0% osmium tetroxide, dehydrated in increasing concentrations of ethanol, and then dehydrated at 31.1°C and 1,072 PSI for 16 min in a critical point dryer. Bladders were quadrisected to reveal the urothelial surface and were mounted on carbon tape-coated stubs and sputter-coated with gold/palladium under argon. Bladders were imaged on a Zeiss Crossbeam 540 FIB-SEM.

### Mouse Model Examining the Effects of *Gardnerella* “Preexposures” on UPEC UTI

Mice were anesthetized with isoflurane and then inoculated intravesically twice, 12 h apart, with either 50 μl of prepared 1 × 10^8^ *Gardnerella* strain JCP8151B inoculum or PBS as a control, as described above. Twelve hours after the second inoculation, mice were inoculated with 50 μl of prepared inoculum containing either 1 × 10^7^ or 1 × 10^6^ UPEC strain UTI89kanR. To monitor acute UPEC infection, urine was collected at 6 and 24 h after UPEC inoculation, and titers were enumerated by serial dilution and plating on LB + kanamycin selective media. A subset of mice was monitored for UPEC persistence in the urinary tract by enumerating UPEC in urine weekly out to 4 weeks postinfection. At 4 wpi, mice were humanely sacrificed by cervical dislocation under isoflurane anesthesia, and bladders were aseptically harvested. Homogenates were prepared in 1 ml of sterile PBS and plated on selective media. Samples with no colonies were plotted at the limit of detection.

### Cytokine and Chemokine Analysis

Cytokine content was measured in mouse serum using the Bio-Plex-Pro Mouse Cytokine 23-Plex, Group I Panel Multiplex Cytokine Bead Kit (Bio-Rad), which quantifies the following 23 cytokines and chemokines: IL-1α, IL-1β, IL-2, IL-3, IL-4, IL-5, IL-6, IL-9, IL-10, IL-12p40, IL-12p70, IL-13, IL-17A, Eotaxin, G-CSF, GM-CSF, IFN-γ, KC, MCP-1, MIP-1α, MIP-1β, RANTES, and TNF-α. The assay was performed according to manufacturer instructions, except using a tenfold less standard and half the amount of coupled beads and detection antibodies indicated in the protocol.

## Data Availability Statement

The data presented in this study are deposited in the GEO repository, accession number GSE203195.

## Ethics Statement

The animal study was reviewed and approved by the Institutional Animal Care and Use Committee (IACUC) of Washington University School of Medicine.

## Author Contributions

NG and VO performed RNA-seq experiments. NG performed preexposure experiments with UPEC, measured cytokines, and performed SEM. CW and EB performed histology and immunofluorescence microscopy. CW enumerated Ki67+ cells. NG, CM, and AL analyzed the data. NG and VO drafted the manuscript. All authors contributed to the article and approved the submitted version.

## Funding

This work was supported by the National Institutes of Health NIAID [R01 AI114635 to AL and R21 AI152049 to AL and NG] and NIDDK [R21 DK092586 to AL, K01 DK110225 to NG, and U54 DK104309 to CM], by the National Science Foundation [Graduate Research Fellowship to VO #DGE-1143954], by the American Heart Association [Postdoctoral Fellowship to NG], and by the Center for Women’s Infectious Disease Research at Washington University School of Medicine in St. Louis [Pilot Research Grant to NG]. This research used the resources of the Herbert Irving Comprehensive Molecular Pathology Shared Resources, funded in part through Center Grant P30 CA013696. Some of the animal studies were performed in a facility supported by the NCRR [C06 RR015502]. The funders had no role in study design, data collection and analysis, decision to publish, or preparation of the manuscript.

## Conflict of Interest

The authors declare that the research was conducted in the absence of any commercial or financial relationships that could be construed as a potential conflict of interest.

## Publisher’s Note

All claims expressed in this article are solely those of the authors and do not necessarily represent those of their affiliated organizations, or those of the publisher, the editors and the reviewers. Any product that may be evaluated in this article, or claim that may be made by its manufacturer, is not guaranteed or endorsed by the publisher.
