# Supplementary material for: Gardnerella Exposures Alter Bladder Gene Expression and Augment Uropathogenic Escherichia coli Urinary Tract Infection in Mice
Source: Front Cell Infect Microbiol. 2022 Jun 16;12:909799. doi: 10.3389/fcimb.2022.909799 (PMC9245024; doi:10.3389/fcimb.2022.909799)
Supplement: Supplementary file 1 [file DataSheet_1.docx]

**
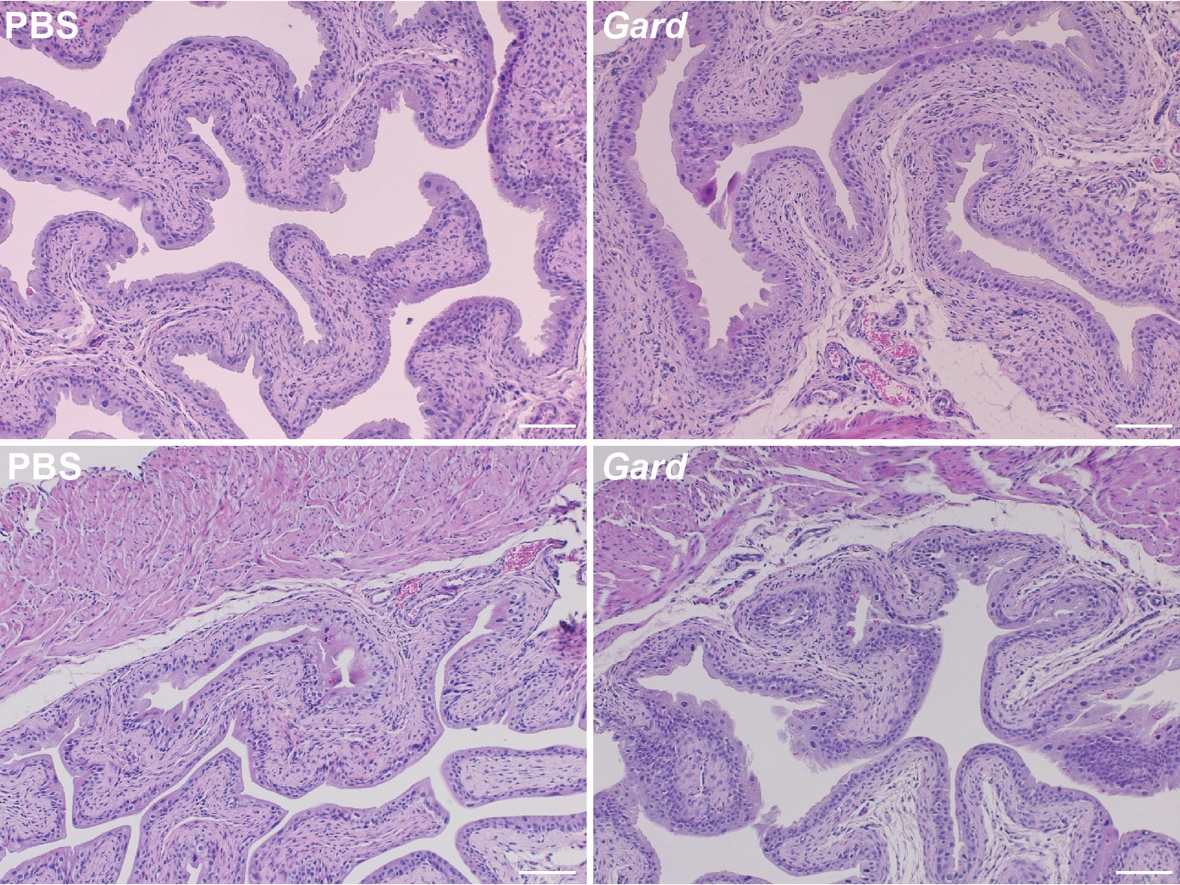
**

**Supplemental Figure 1. *Gardnerella* exposure does not cause robust histological inflammation.** H&E staining of bladder sections from mice exposed to PBS or *Gardnerella*, using the same experimental timeline shown in Figure 1A. Scale bars = 100 μm.

**
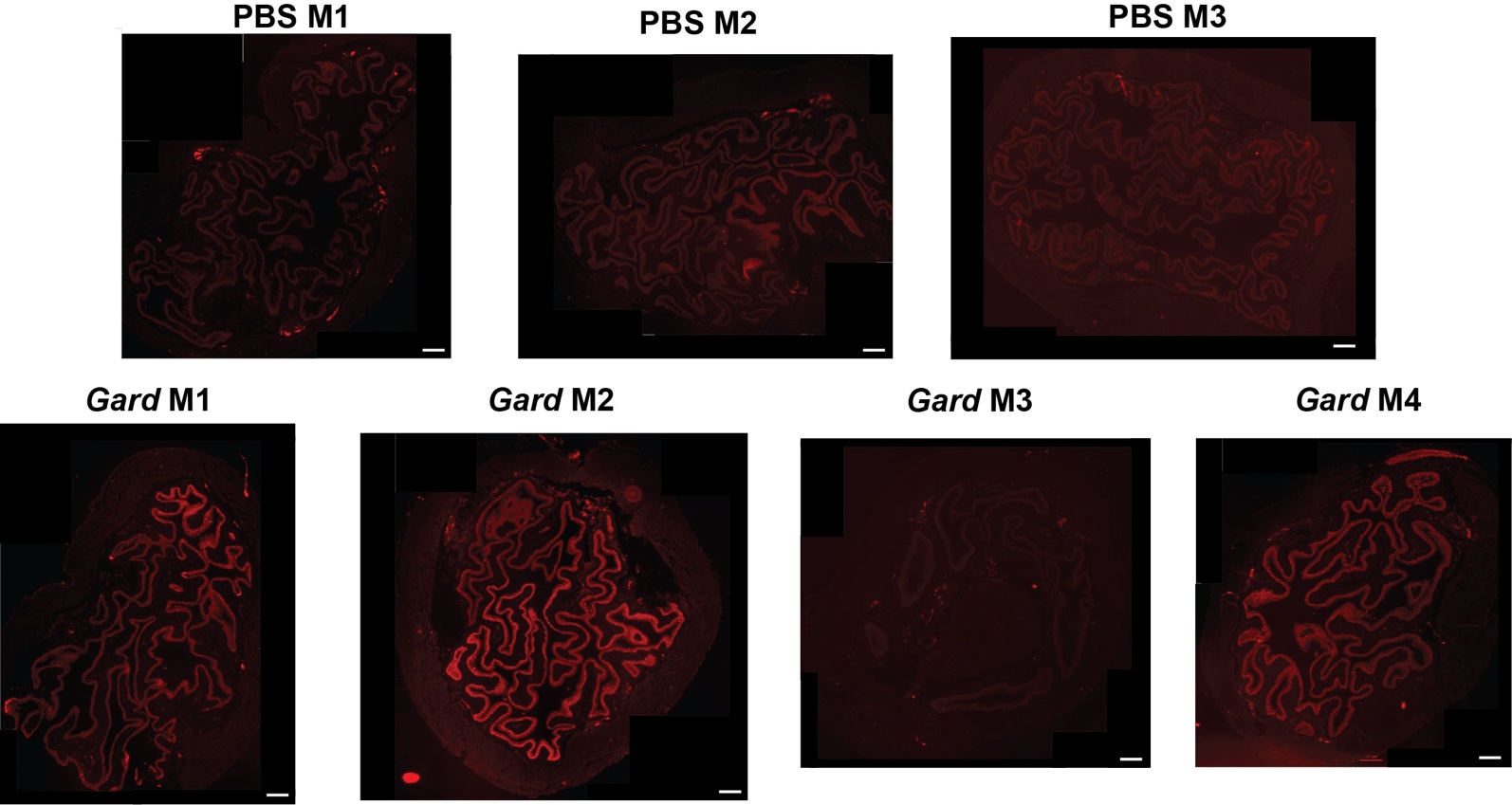
**

**Supplemental Figure 2. Immunofluorescent detection of Keratin 6 protein in bladder sections.** Images were taken with a 10x objective and images were stitched together to generate a composite panoramic image of the entire bladder section. The same microscope settings (laser power, gain) were used to acquire each image to allow side-by-side comparisons. Scale bars = 200 μm


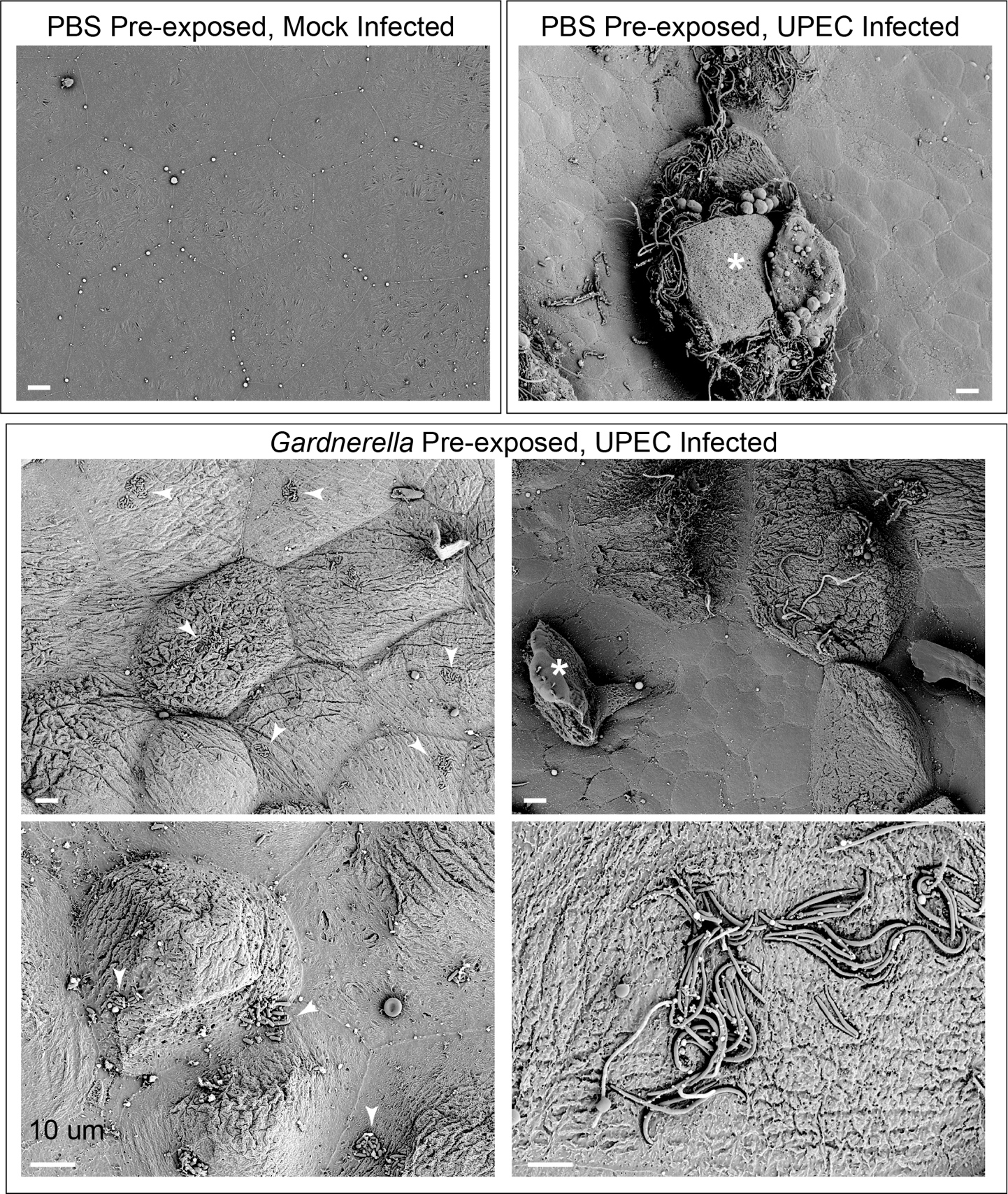


**Supplemental Figure 3**. SEM images of bladders from mice pre-exposed to PBS (top) or *Gard* (bottom). Bladders were collected 6 h after infection with UPEC (top, right and bottom, both) of mock infection with PBS (top left). Asterisks mark exfoliating umbrella cells. Arrowheads point to collections of adherent bacillary UPEC, as described in the text. Scale bars = 10 μm.

**Supplemental Figure 4**. Acute UPEC titers in urine collected 24 hpi in the pre-exposure model. Each dot represents and individual mouse (n = 5 per group). Box plot denotes the 25^th^ and 75^th^ percentile with a line at the median and whiskers from min. to max.

**Supplemental Figure 5. Serum pro-inflammatory cytokines were not altered by *Gardnerella* exposure.** Mice were exposed twice to PBS or *Gardnerella*, as shown Figure 1A, and serum was collected 12 h after the second exposure. This represents the same time point at which mice received an inoculation of UPEC in the “pre-exposure” model, but mice did not receive any UPEC inoculations in these experiments. Cytokines were measured using a Bio-Plex assay. Data points are each from a single mouse (n = 10 mice per group) and geometric means are plotted. Mann-Whitney tests did not detect significant differences between PBS and *Gard*.


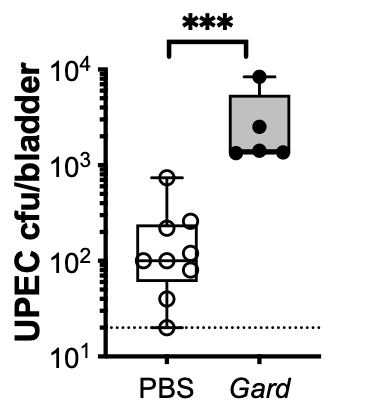


**Supplemental Figure 6.** UPEC titers in bladders from mice that resolved UPEC bacteriuria, thereby indicative of intracellular reservoirs. *** P < 0.001 Mann-Whitney U test. Each dot represents an individual mouse (PBS n = 9, *Gard* n = 5). Data are from the same experiment and are a subset of what is shown in Figure 4F. Dotted line denotes the limit of detection. Box plot denotes the 25^th^ and 75^th^ percentile with a line at the median and whiskers from min. to max.

**Key Reagents and Resources**

| **REAGENT or RESOURCE** | **SOURCE** | **IDENTIFIER** |
| --- | --- | --- |
| **Antibodies** | | |
| Chicken Polyclonal Anti-Keratin 5 | Biolegend | Cat#905901 |
| Rabbit Polyclonal Anti-Krt6A | LSBio | Cat#LS-B12036-100 |
| Mouse Monoclonal Anti-Cytokeratin 20 | AgilentDako | Cat# M701929-2 |
| Goat Polyclonal Anti-p63 | R&D Systems | Cat#AF1916 |
| Rabbit Polyclonal Anti-Ki67 | Abcam | Cat#ab15580 |
| Alexa Fluor 488 Donkey Anti-Mouse IgG | Jackson Immunoresearch | Cat#711-545-150 |
| Alexa Fluor 488 Donkey Anti-Chicken IgG | Jackson Immunoresearch | Cat#703-545-155 |
| Cy3 Donkey Anti-Rabbit IgG | Jackson Immunoresearch | Cat#711-165-152 |
| Alexa Fluor 647 Donkey Anti-Goat IgG | Jackson Immunoresearch | Cat#705-605-003 |
| Alexa Fluor 647 Donkey Anti-Mouse IgG | Jackson Immunoresearch | Cat#715-605-150 |
| **Mice** |  |  |
| C57BL/6NCrl females | Charles River | Strain code 027 |
